# The Fitting of the OJ Phase of Chlorophyll Fluorescence Induction Based on an Analytical Solution and Its Application in Urban Heat Island Research

**DOI:** 10.3390/plants13030452

**Published:** 2024-02-03

**Authors:** Tongxin Shi, Dayong Fan, Chengyang Xu, Guoming Zheng, Chuanfei Zhong, Fei Feng, Wah Soon Chow

**Affiliations:** 1 The Key Laboratory for Silviculture and Conservation of Ministry of Education, College of Forestry, Beijing Forestry University, Beijing 100083, China; shitongxin66@163.com (T.S.);; 2 Yi Zong Qi Technology (Beijing) Co., Ltd., Beijing 100095, China; 3 Institute of Forestry and Pomology, Beijing Academy of Agriculture and Forestry Sciences, Beijing 100093, China; 4 Division of Plant Sciences, Research School of Biology, The Australian National University, Acton, ACT 2601, Australia

**Keywords:** connectivity among PSII complexes, differential equation of Q_A_ reduction kinetics, functional absorption cross-section of PSII, heat stability of PSII, UHI

## Abstract

Chlorophyll (Chl) fluorescence induction (FI) upon a dark–light transition has been widely analyzed to derive information on initial events of energy conversion and electron transfer in photosystem II (PSII). However, currently, there is no analytical solution to the differential equation of Q_A_ reduction kinetics, raising a doubt about the fitting of FI by numerical iteration solution. We derived an analytical solution to fit the OJ phase of FI, thereby yielding estimates of three parameters: the functional absorption cross-section of PSII (*σ*_PSII_), a probability parameter that describes the connectivity among PSII complexes (*p*), and the rate coefficient for Q_A_^−^ oxidation (*k*_ox_). We found that *σ*_PSII_, *p*, and *k*_ox_ exhibited dynamic changes during the transition from O to J. We postulated that in high excitation light, some other energy dissipation pathways may vastly outcompete against excitation energy transfer from a closed PSII trap to an open PSII, thereby giving the impression that connectivity seemingly does not exist. We also conducted a case study on the urban heat island effect on the heat stability of PSII using our method and showed that higher-temperature-acclimated leaves had a greater *σ*_PSII_, lower *k*_ox_, and a tendency of lower *p* towards more shade-type characteristics.

## 1. Introduction

Chlorophyll (Chl) fluorescence induction (FI) has been extensively analyzed to investigate the initial events of electron transfer and energy conversion through PSII in plants, algae, and cyanobacteria [1,2,3,4,5,6,7]. During a dark–light transition, the FI curve shows a complicated rise, with several phases distinguished as O–J–I–P transients [3]. FI after the J point has been regarded as being linked to electron transfer and fluorescence quenching of different origins beyond Q_A_ (the primary quinone acceptor of PSII). By contrast, the OJ phase is relatively simple, mainly reflecting the accumulation of reduced Q_A_. There is experimental and theoretical evidence that the OJ phase is the ‘photochemical phase’ connected with primary photochemistry [8]. Thus, numerous efforts have been made to fit the OJ curve to deduce kinetic and structural parameters such as the functional absorption cross-section of PSII and its connectivity [3,9,10,11], principally based on the “exciton/radical-pair equilibrium” model [12]. Such information is important for studies regarding the estimation of the fraction of open PSII centers [13], the charge-recombination of S_2_Q_A_^−^ [14], the photoprotective role of non-photochemical quenching [15], and the photosynthetic efficiency associated with crop yield [16].

Equations based on the “exciton/radical-pair equilibrium” model have been established for single-turnover situations, which have catalyzed the birth of a new approach to LIFT/FRR fluorescence technology [1,2]. In addition, the OJ phase is considered informative also for the multiple-turnover situation [3]. For example, Morin [4] observed that the amplitude of the OJ phase depends strongly on the number of photons absorbed by the sample. Theoretically, the OJ rise should be exponential if one photon absorbed by the antenna system results in the transfer of one electron in an open reaction center (RC) to Q_A_, thereby leading to the closure of the PSII trap once Q_A_ is reduced. The reduction in Q_A_ raises the fluorescence yield from the O point to the J point in about 2 ms. However, it was found that the OJ rise is a sigmoidal shape instead of an exponential one, explicable by several hypotheses (PSII heterogeneity, energetic connectivity of PSII, quenchings/annihilations of different origins, overlap between thermal phase and photochemical phase, Y_z_^+^ quenching, conformation change, etc.) [5,6,7,8,9,10,11], though there exists no general consensus in this regard.

Despite the existing debate, a view holds that PSII RCs are embedded in a large pigment matrix with a probability of energy transfer from a closed PSII to an adjacent open RC [5], consequently leading to the sigmoidal OJ fluorescence rise. Specifically, the initial fluorescence rise is slowed because it is easy for an exciton that visits a closed PSII RC to be transferred to its neighboring open PSII RCs where the fluorescence is quenched by oxidized Q_A_ such that the fluorescence yield is close to the *F*_o_ level that exists when all PSII RCs are open. With the increase in closed PSII RC content, the probability of finding an adjacent open center decreases quickly; therefore, the fluorescence yield approaches the *F*_m_ level. This model has been comprehensively verified by the OJ fitting technique [12,13], the LIFT/FRR technique [1,2,14], fluorescence lifetime analysis [15], exciton annihilation and time-resolved photoelectric experiments [16], and CD spectrum analysis [17]. It was found that the probability *p* (called connectivity parameter) of the excitation energy transfer from a closed RC to a neighboring RC was generally ~0.55, with the corresponding *J* (sigmoidicity parameter) ~1.5 [18], corresponding to 3–5 PSII units being excitonically connected [19,20]. On the other hand, an updated single-turnover flash-induced O_2_ evolution method does not support such a model; instead, it has been argued that PSII antennae are not energetically connected after a few milliseconds of illumination [21,22]. As such, the hypothesis that PSII antennae are excitonically connected is facing challenges.

Two arguments were presented to question customary OJ fitting methods. The first is that all current fitting methods are based on a numerical iteration process because there seems to be no analytical solution to the differential equation of Q_A_ reduction kinetics, which are derived from the “exciton/radical-pair equilibrium” model [20]. It is therefore argued that the adjustment of parameters can predict rather similar FI curves independent of the actual structure of the light-harvesting apparatus [21]. Secondly, there seems to be some uncertainty as to the chlorophyll fluorescence yield corresponding to fully closed PSII traps. The maximum yields of chlorophyll fluorescence as induced by a single-turnover saturating flash at/before the J point (*F*_mj_) is always lower than the *F*_m_ level as induced by a multiple-turnover saturating pulse (>200 ms) [8,23]. It was also found that maximum yields of chlorophyll fluorescence obtained with the prototype LIFT/FRR instrument were smaller than those obtained from the same leaf using the saturating multiple-turnover pulse in the pulse amplitude modulation technique (PAM, [24]). Such a difference between *F*_mj_ and *F*_m_ was initially attributed to quenching by oxidized PQ [8,25] and lately to the difference between the charge-separated closed state and the light-adapted charge-separated state [26]. Since *F*_mj_ reflects the fully-reduced Q_A_ state and participates in the OJ fitting, it is critical to determine *F*_mj_ appropriately at the OJ phase [2,13,27,28,29]. Unfortunately, for a commercial instrument like a Handy plant efficiency analyzer (PEA), no single-turnover flash is available during the continuous recording of fluorescence on the transition from dark to light, resulting in only a semi-empirical estimation of the functional absorption cross-section of PSII and its connectivity [12,30].

In the present study, we provide an analytical solution to the differential equation of Q_A_ reduction kinetics, hence minimizing the uncertainties of parameter estimation. Additionally, we seek a statistical solution to find *F*_mj_, based on the prediction by the “exciton/radical-pair equilibrium” model that different intensities of the excitation light should yield the same PSII functional absorption cross-section, which has already been demonstrated by the LIFT/FRR techniques [1]. Then, based on the analytical formulae, we investigated the changes of fitted PSII functional absorption cross-section, connectivity, and Q_A_ oxidation rate coefficients by fitting the FI of different time periods of illumination up to 1 ms. The major aims of the present study are to (1) provide an analytical solution to the differential equation of Q_A_ reduction kinetics, (2) attempt to resolve the controversy over the PSII connectivity, and (3) provide a case study on the UHI (urban heat island) effect on the heat stability of PSII by our method. Since changes in plant morphology initiated by high ambient temperature and by vegetation shade are very similar [31,32], we hypothesize that higher UHI leads to a lower rate of Q_A_^−^ oxidation, higher functional absorption cross-section of PSII, and/or lower connectivity among PSII complexes.

## 2. Results

**Figure 1:**
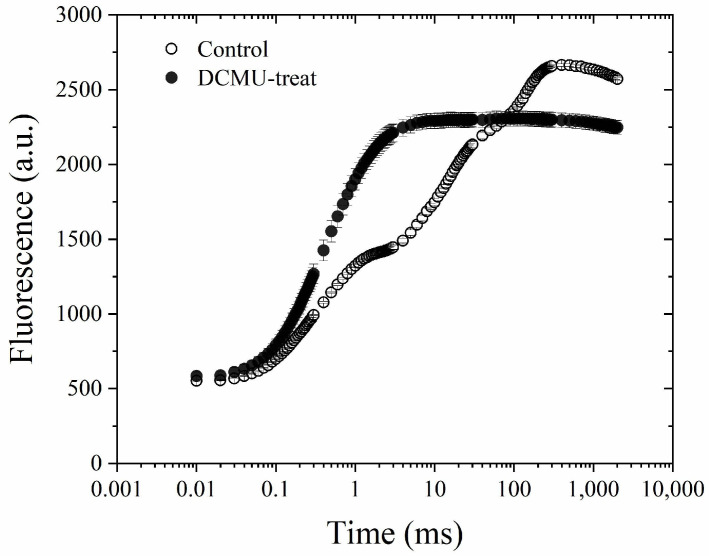
Chlorophyll *a* fluorescence transients of spinach leaves treated with DCMU or untreated. Excitation light intensity, at the leaf surface, was 3400 μmol photons m^−2^ s^−1^. Data are plotted on a semi-logarithmic time scale. *n* = 3, bar = ±1 s.e.

At room temperature, the initial fluorescence rise kinetics during the first 100–150 μs of illumination were the same in both untreated and DCMU-treated samples (Figure 1). However, the rise time of the fluorescence transient curve, in the presence of DCMU, was shorter than that of the O–J phase in the untreated sample. This result is similar to that of Schansker et al. [10]. *F*_m_ measured with DCMU was smaller than that without DCMU, probably due to the non-photochemical quenching by PQ [33] or abolition of variable fluorescence from PSI [27].

**Figure 2:**
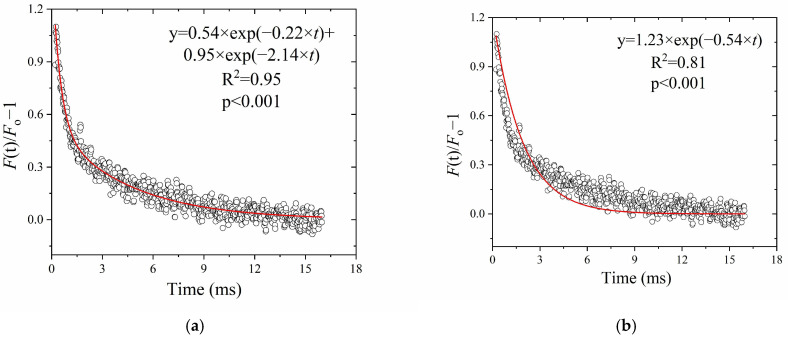
The *k*_ox_′ obtained from Q_A_^−^ re-oxidation kinetics after a single-turnover flash. (**a**) Two components of decay; (**b**) one component of decay. The values of *F*(t)/*F*_o_ − 1 are the average of three repetitions.

Q_A_^−^ re-oxidation kinetics after a single-turnover flash can be fitted by a first-order rate law, with two (Figure 2a) or one component(s) (Figure 2b). The fitting by the two-component first-order reaction was better than by one component. However, we found that, for nonlinear curve-fitting in the least-squares manner in the present practice, multiple components could not be distinguished. As such, the overall oxidation rate coefficient (*k*_ox_′, ms^−1^) fitted by one exponential component in the single-turn-over situation (Figure 2b), was compared to the OJ fitting output (*k*_ox_, ms^−1^) in the following results.

**Figure 3:**
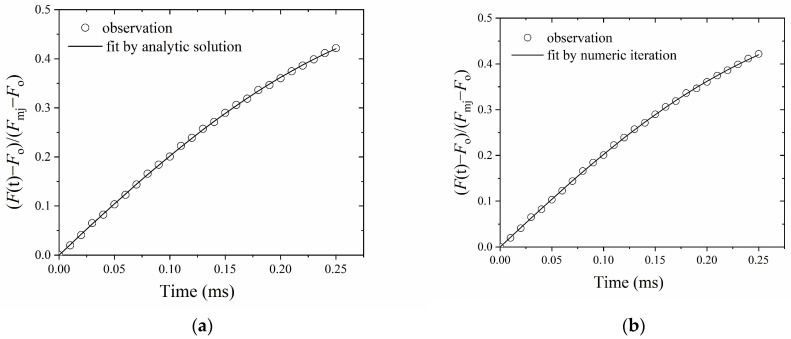
Parameters estimated by an analytical solution (**a**) and numerical iteration (**b**). In (**a**): *σ*_PSII_ = 4.33 (nm^2^), *p* = 0.49, *k*_ox_ = 0.48 (ms^−1^), RMSE = 0.026. In (**b**): *σ*_PSII_ = 4.20 (nm^2^), *p* = 0.48, *k*_ox_ = 2.10 (ms^−1^), RMSE = 0.026. Fitting was conducted on the data points up to 0.25 ms of exposure to red light of irradiance 3400 μmol m^−2^ s^−1^. It is worth noting that in this measurement, *k*_ox_ obtained by analytical solution (0.48 ms^−1^) is close to *k*_ox_′ (0.54 ms^−1^) in Figure 2b. The values of (*F*(t)/*F*_o_)/(*F*_mj_ − *F*_o_) are the average of three repetitions.

Figure 3a shows the fitting by an analytical solution with a home-made Matlab (Matlab, R2010b; the MathWorks, Natick, MA, USA) code. Figure 3b shows the fitting by a numerical iteration method with a homemade visual basic (Microsoft Corp., Redmond, WA, USA) code. The fitting parameter outputs by an analytical solution and a numerical iteration procedure differed little from each other, despite the *k*_ox_ being lower in the analytical solution probably due to an insufficiently short integration interval time in our visual basic code. Hereafter, we used the analytical solution to fit the OJ curves.

**Figure 4:**
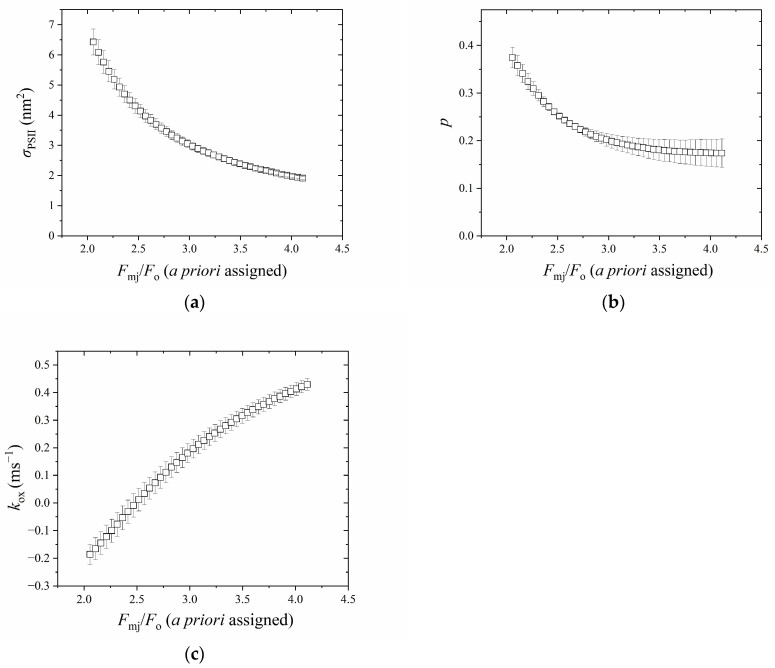
The relationship between a priori assigned *F*_mj_/*F*_o_ and fitted *σ*_PSII_ (**a**), fitted *p* (**b**), and fitted *k*_ox_ (**c**). The above parameters were calculated with 1 ms data. *n* = 3, bar = ±1 s.e.

It can be seen from Figure 4 that *σ*_PSII_, *p* and *k*_ox_ varied greatly with a priori assigned *F*_mj_/*F*_o_. *σ*_PSII_ decreased from 6.42 to 1.90 nm^2^ (Figure 4a), *p* decreased from 0.37 to 0.17 (Figure 4b), and *k*_ox_ increased from −0.18 to 0.42 ms^−1^ (Figure 4c), with the increase in *F*_mj_/*F*_o_ from 2.3 to 4.2.

**Figure 5:**
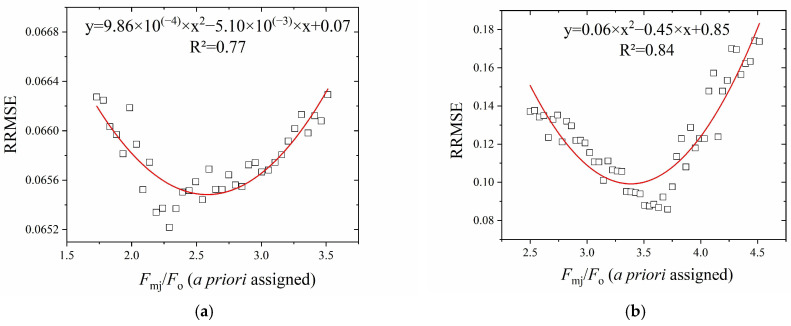
The relation of RRMSE of *σ*_PSII_ to a priori assigned *F*_mj_/*F*_o_. (**a**) Control; (**b**) DCMU-treatment. These curves were empirically fitted by a basic binomial function. By setting the derivative of the function to zero, the lowest RRMSE value is obtained. In this way, the lowest RRMSE value corresponded in (**a**) to *F*_mj_/*F*_o_ = 2.58 and in (**b**) to *F*_mj_/*F*_o_ = 3.39. Fitting was conducted on fluorescence data up to 0.20 ms of illumination.

Since the estimation of parameters (*σ*_PSII_, *p*, *k*_ox_) in Equation (6) largely depended on *F*_mj_/*F*_o_ (Figure 4), we sought a statistical solution to find the *F*_mj_ during the O-J phase in the absence of a saturating pulse for a Handy PEA type fluorimeter. Figure 5 shows that there existed a minimum RRMSE of *σ*_PSII_ along the gradient of excitation light intensity (3400, 3300, 3200, and 3100 μmol m^−2^ s^−1^) for both control and DCMU treatment. The *F*_mj_ corresponding to the *F*_mj_/*F*_o_ at the minimum RRMSE was supposed to be the best estimation, according to the results of Kolber et al. [1].

**Figure 6:**
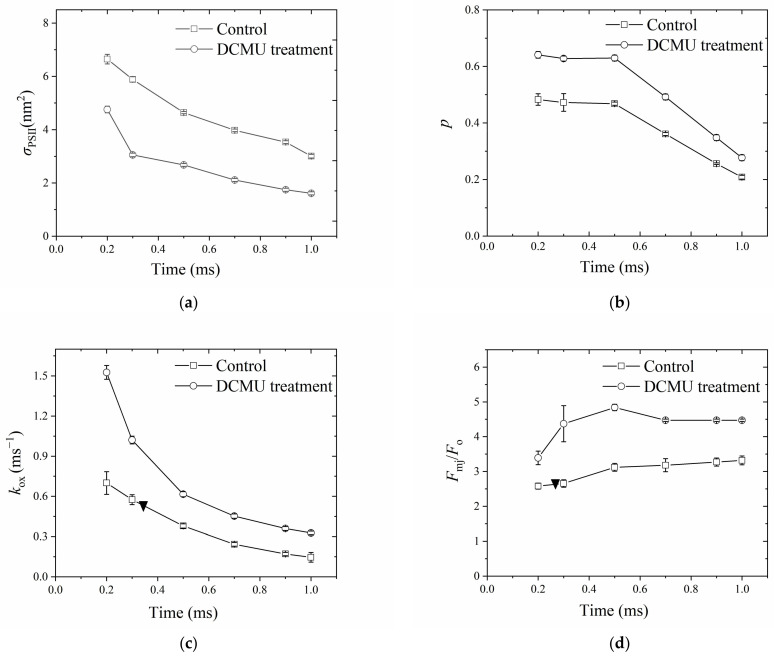
The values of *σ*_PSII_ (**a**), *p* (**b**), *k*_ox_ (**c**), and *F*_mj_/*F*_o_ (**d**) as a function of time of illumination in the absence or presence of DCMU. The solid inverted triangle in Panel (**c**) represents the situation where *F*_mj_/*F*_o_ is 2.4, obtained by fitting with two components of decay in Figure 2a. In Panel (**d**), the solid inverted triangle represents the situation where *k*_ox_ is 0.54 ms^−1^, obtained by fitting with one component of decay in Figure 2b. However, it is worth noting that after 0.50 ms, the DCMU treatment could not yield *F*_mj_ by the statistical solution, for some reason. Hence, the *F*_mj_ after 0.50 ms was assumed to be the *F*_m_ at the P point. *n* = 3, bar = ±1 s.e.

The OJ fluorescence data of control and DCMU-treatment were fitted for different illumination times (0.20, 0.30, 0.50, 0.70, 0.90, and 1.00 ms); the estimation of *σ*_PSII_, *p*, *k*_ox_, and *F*_mj_/*F*_o_ are shown in Figure 6. For *σ*_PSII_ (Figure 6a), with the increase in illumination time, the fitted value decreased from 6.64 to 3.00 nm^2^ in the control, while in the DCMU-treatment, it decreased from 4.75 to 1.60 nm^2^. For *p* (Figure 5b), with the increase in illumination time, the fitted value decreased from 0.48 to 0.21 in the control, while in the DCMU-treatment, it decreased from 0.64 to 0.28. It is worth noting that *p* did not change up to 0.5 ms (Figure 6b). For *k*_ox_ (Figure 6c), with the increase in illumination time, the fitted value decreased from 0.70 to 0.15 ms^−1^ in the control, while in the DCMU-treatment, it decreased from 1.53 to 0.33 ms^−1^. In Figure 2b, the *k*_ox_ fitted by a one-exponential component, representing the overall oxidation rate coefficient, was 0.54 ms^−1^ and the corresponding illumination time was about 0.34 ms, as demonstrated by the solid inverted triangle in Figure 6c.

Figure 6d shows the relation of *F*_mj_/*F*_o_ to the time of illumination, where *F*_mj_/*F*_o_ was determined by the statistical method. With the increase in illumination time, the estimated *F*_mj_/*F*_o_ increased slightly from 2.17 to 3.12 for the control. For DCMU-treatment, with the increase in illumination time from 0.20 to 0.50 ms, the estimated *F*_mj_/*F*_o_ increased greatly from 3.30 to 4.84. However, it is worth noting that after 0.50 ms, the DCMU-treatment could not yield *F*_mj_ by the statistical solution. This is due to the fact that in the DCMU-poisoned samples, fluorescence reached the *F*_m_ level very fast and before 2 ms [11]; as such, the statistical solution could not find the lowest RRMSE after 0.50 ms. The solid inverse triangle in Figure 6d represents the location of *F*_mj_/*F*_o_ = 2.40, which is the total amplitude as fitted by the two components of decay in Figure 2a. The corresponding illumination time was 0.26 ms.

**Figure 7:**
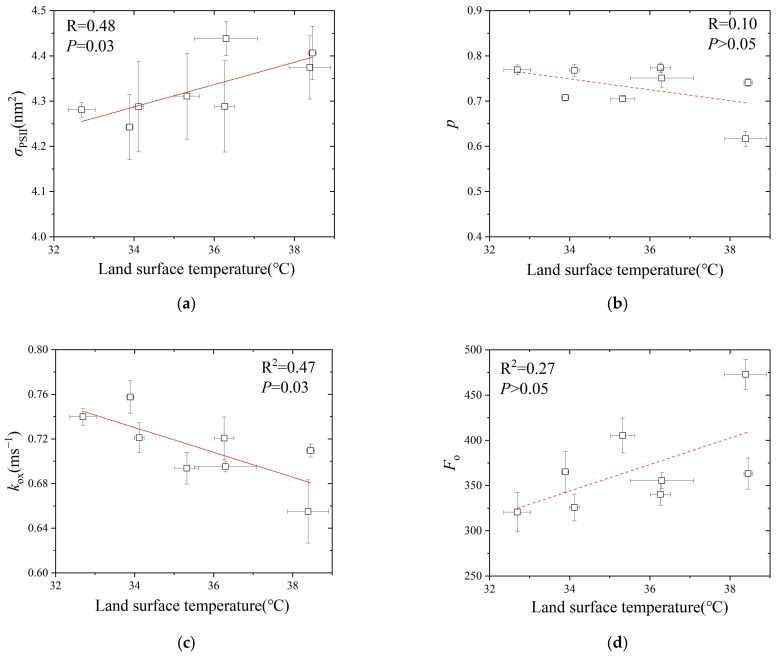
The relationship between land surface temperature and *σ*_PSII_ (**a**), *p* (**b**), *k*_ox_ (**c**), and *F*_o_ (**d**) of eight sampling sites in Beijing. In (**a**): R^2^ = 0.48, *P* = 0.03; In (**b**): R^2^ = 0.10, *P* > 0.05; In (**c**): R^2^ = 0.47, *P* = 0.03; In (**d**): R^2^ = 0.27, *P* > 0.05. *n* = 4, bar = ± 1 s.e.

Among the eight sites, the average land surface temperature in July (2020–2022) ranged from 32.7 °C to 38.5 °C. The OJ fluorescence data were fitted before illumination times of 0.30 ms; the estimation of *σ*_PSII_, *p*, *k*_ox_, and *F*_o_ are shown in Figure 7. For *σ*_PSII_ (Figure 7a), with the increase in land surface temperature, *σ*_PSII_ tended to increase from 4.24 to 4.43 nm^2^. For *p* (Figure 7b), with the increase in land surface temperature, *p* tended to decrease from 0.77 to 0.61. For *k*_ox_ (Figure 7c), with the increase in land surface temperature, *k*_ox_ decreased linearly from 0.76 to 0.65 ms^−1^. For *F*_o_ (Figure 7d), with the increase in land surface temperature, *F*_o_ tended to increase from 320 to 472 (rel.).

## 3. Discussion

### 3.1. The Mechanism(s) Underlying OJ Rise Is(Are) Complicated and Highly Controversial

One of the key arguments against the use of the differential equation based on an exciton/radical-pair model with energy transfer between photosynthetic units (Equation (3)) is that different combinations of values of parameters in a numerical iteration can lead to a similar fitting output, mainly due to there being no analytical solution to the Q_A_ reduction differential equation. However, we have shown in the present study that the fitting parameter outputs by an analytical solution and a numerical iteration procedure differed little from each other, despite the *k*_ox_ being lower in the analytical solution. Thus, we need not worry about the suggestion by Oja and Laisk [11] that a numerical iteration does not give reliable parameter values.

Secondly, the critical role of *F*_mj_ in determining *σ*_PSII_ and p could be seen in Figure 4a–c. The *F*_mj_, as determined by the statistical solution, was found to be lower than *F*_m_ at the P point, consistent with numerous studies showing that *F*_mj_ as induced by a single-turnover flash is lower than *F*_m_ by about 35–50% by the LIFT/FRR technique and PAM technique [1,2,13,24,34,35,36]. A lower *F*_mj_ relative to *F*_m_ may suggest a second hypothetical quencher, R instead of Q_A_, which quenches fluorescence prior to the P peak [37]. Many hypotheses regarding various quenching mechanisms such as oxidized PQ pool [2,8] and the presence of Q_B_ quenching [8] have been presented. With the aid of a statistical solution, it is interesting to find that the lowering of *F*_mj_ relative to *F*_m_ has largely been diminished in the DCMU treatment (Figure 6d). This suggests that such quenching is not related to the oxidized PQ, consistent with the results of Tóth et al. [38], probably due to Q_B_ quenching as proposed by Schreiber et al. [39], since DCMU irreversibly occupies the Q_B_-pocket [40]. We also observed that DCMU treatment caused quenching of *F*_mj_ to some extent before 0.5 ms compared with the *F*_mj_ later at the J point (Figure 6d), similar to that observed by Schreiber and Krieger [39], attributable to a transient type of nonphotochemical quenching, probably due to nonradiative recombination and/or triplet formation [41], the presence of oxidized non-heme iron [42], or the inactive branch of PSII [41]. More recently, it has been demonstrated that the whole transient from the I point to the P point is due to variable fluorescence emission from PSI [27,43], consistent with its inhibition by DCMU. Interestingly, no substantial quenching of *F*_mj_ was observed beyond 0.5 ms despite a decrease in *k*_ox_ in the presence of DCMU after 0.5 ms (Figure 6c,d) during a dark–light transition period; this indicates that such quenching before 0.5 ms is related to non-radiative P680^+^ charge-recombination [44]. This is supported by the delayed luminescence studies [45] if the 40 μs delayed light emission signal is regarded as a sign of a closed RC [10,45]. The results support the notion that PSII RCs are almost entirely closed at the J point [13,25,37,41,46,47,48,49]. Further, Sipka et al. [26] pointed out that the difference between *F*_mj_ and *F*_m_ is actually ascribed to the difference between the charge-separated closed state and the light-adapted charge-separated state. It is worth noting that one argument states that PSII RCs are almost entirely closed at the P instead of the J point, as the commonly-used *F*_v_/*F*_m_ is in agreement with the maximum photochemical yield of oxygen evolution or carbon assimilation [50,51]. However, *F*_v_/*F*_m_ may not represent the maximum photochemical yield as discussed by Vredenberg et al. [52] and Sipka et al. [26] and in intact leaves, leaf structure as well as fluorescence re-absorption can complicate the situation further [53,54,55].

It has long been proposed that a significant portion of excitation energy absorbed in the antennae of closed centers can be transferred to remaining open centers [18]. Our fitting, up to 0.50 ms of illumination time, showed that *p* ≈ 0.5, consistent with previous studies, either by LIFT/FRR [1,2], by PAM with single-turn-over flash [13,56], or by semi-empirical OJ fitting from a Handy PEA type fluorescence meter [12]. However, our fitting also showed that “apparent” *p* decreased if the illumination time was longer than 0.50 ms (Figure 6b), supporting the claim by Oja and Laisk [21] that it is “an internal, dark–light adjustable state of PSII” during the “ripening” phase [11]. Our fitting output is also consistent with the observation that *p* showed dynamic changes during the dark–light transition when Q_A_ is gradually reduced, due to the “active islet”/domain effect [57]. Thus, some researchers have suggested that the *p* parameter should be calculated when the fraction of reduced Q_A_ is smaller than 0.6 [58,59]. The typical fitting of OJ, as well as LIFT/FRR, is based on a fixed illumination time (2 ms for OJ, or 100–400 μs for LIFT/FRR) [1,2,12,13,56]; therefore, it does not capture the dynamics of *p* during the dark–light transition period. Such dynamic *p* during light induction, as revealed by the present study, therefore, can be one reason for the dispute about PSII connectivity between different research groups.

Meanwhile, our fitting also showed that the functional absorption cross-section of PSII (*σ*_PSII_) decreased with the increase in illumination time (Figure 6a); this is possibly attributed to decreased primary charge-separation rate as the consequence of reduced Q_A_, the decrease in connectivity, or decreased efficiency of excitation transfer from the pigment bed to the PSII reaction center. Such *σ*_PSII_ dynamics during the dark–light transition period can explain how the LIFT technique shows a higher *σ*_PSII_ than estimates from intro methods on the Chl *b*-less barley mutant and Chl *b*-depleted Arabidopsis mutant; low-light acclimated barley has noticeably lower values for *σ*_PSII_ and the optical absorption cross-section than high-light-grown barley [2], when the fitting is based on a fixed illumination time. It is, therefore, recommended that *σ*_PSII_ can only be compared between species/treatments when other effects are minimized. In fact, it has been found that the primary charge separation reaction rate decreases by about 2–5 fold after the reduction in Q_A_ [39,60,61,62,63]. On the other hand, energy-dependent non-photochemical quenching NPQ (qE) should not exert such an effect because it occurs by an order of magnitude more slowly than *σ*_PSII_ dynamics.

Treatment with DCMU gave a p-value greater than that of the control, decreasing similarly to the control (Figure 6b). Meanwhile, the DCMU treatment gave a *σ*_PSII_ value smaller than that of the control (Figure 6a). This observation suggests that *σ*_PSII_ and *p* estimation are affected by treatment with DCMU, inconsistent with the semi-empirical OJ fitting output by Strasser et al. [3] but consistent with the observation of Joliot and Joliot [19] who found *p* values of ≈0.7 in subfreezing samples when the electron transfer from Q_A_ to Q_B_ is greatly retarded by low temperature. This observation is plausible as DCMU treatment may (1) induce a thermal component at the OJ phase [10]; (2) effectively decrease the membrane electric potential [64], primary charge-separation rate [39], and the efficiency of energy transfer between chlorophyll molecules; or (3) change the configuration of PSII complexes and the organization of thylakoid membranes [65]. All these factors can affect the estimation of *σ*_PSII_ and *p* according to the “exciton/radical-pair equilibrium” model [20].

Interestingly, DCMU treatment also led to a higher *k*_ox_ than the control (Figure 6c), indicating the occurrence of enhanced radiative RC charge recombination under DCMU treatment. In fact, it was found that the rate constant of charge recombination between Pheo^−^ and P680^+^ in closed PSII reaction centers is three times higher than that of open ones [66]. The *k*_ox_ decreased with the increase in illumination time for both DCMU and control treatments, suggesting that other Q_A_ oxidation processes (e.g., cyclic electron flow in PSII [67]) with slower rate constants gradually dominate in *k*_ox_ estimation. However, we must acknowledge that the underpinning mechanism(s) for *σ*_PSII_ and *p* dynamics during the dark–light transition period are complicated and remain unresolved currently [18,25].

### 3.2. The Question on ‘the Absence of Excitonic Connectivity between PSII Units’ [11]

The “internal, dark-light adjustable state of PSII” [11] could be explained from a different perspective. After exposure to continuous high light, an intense competition for the excitation energy by various energy dissipation pathways such as carotenoid triplet quenching in the antenna, light-independent constitutive excitation dissipation (e.g., charge recombination) in RC [represented by Y (NO) = *F*/*F*_m_], and PSII cyclic electron transport would occur, during or immediately after a subsequent single turn-over flash (STF). For example, carotenoid triplet quenching annihilates the excitation energy that otherwise could move to an open RCII and can be up-regulated under anoxic conditions and continuous high light [68,69]. Another study has shown that from the first seconds to one min following illumination of a control sample at 1500 µmol m^−2^ s^−1^, the P700^+^ kinetics area (a rapid, empirical, whole-tissue, and non-intrusive measurement of the fraction of open PSII, induced by a flash that is only just saturating) is much less than that obtained after sufficient dark relaxation [70]; the decrease is attributable to various energy dissipation pathways that compete for energy from the flash that is only just saturating, before they relax sufficiently in darkness. That is, there is little energy transfer from closed to open PSII centers because this energy-transfer pathway cannot compete against other pathways of energy loss in closed PSII centers and antennae, regardless of the extent of excitonic connectivity among closed and open PSII centers. Obviously, the PSII excitonic connectivity and functional absorption cross-section estimations can only be compared across species or treatments when other pathways of energy dissipation are not too dominant/variable. For a Handy PEA type fluorescence meter, we hence recommend that fitting of data points before 0.30 ms is a good option because it approximates the STF situation (Figure 6) and is consistent with the theoretical deduction [12]. It is worth noting that the *F*_mj_/*F*_o_ at/before 0.30 ms is about 2.5, close to the value of the PSII-closed state induced by the first STF (*F*1/*F*_o_) [26]. This suggests the local electric-field transients, dielectric relaxation processes, and/or conformational changes (which lead to the light-adapted charge-separated state, [26]) have minimal impact on the photochemical phase of the fast Chl-*a* fluorescence induction kinetics before 0.3 ms, in the absence of PSII inhibitor and under physiologically relevant conditions. Furthermore, it is possible that under continuous light, the single-turnover flash-induced O_2_ evolution method cannot quantify the fraction of open PSII RC reliably because other O_2_ uptake pathways intertwine with PSII O_2_ evolution [67,71,72,73].

### 3.3. What Other Factors Might Influence the OJ Phase of Chlorophyll Fluorescence Induction?

PSI fluorescence during the fluorescence transient is practically constant [74,75,76] and its contribution to the total fluorescence signal at room temperature is relatively low [77,78]. However, Schreiber [27] reported evidence that supports the notion that the whole I2-P transient is due to variable PSI fluorescence. Nevertheless, PSI fluorescence is unlikely to contribute to the O-J phase of fluorescence induction.

### 3.4. The Application of the Model for Urban Heat Island Research—A Case Study

In most of the large cities, the temperature at the heart or the center of the city is noted to be higher than its surroundings or the suburban area, mainly due to lower evapotranspiration, high absorption of solar radiation, hindrance to the flow of air, and high heat release by people [79]. The phenomenon is called the urban heat island (UHI) effect [80]. The performance and services of trees may be compromised by UHI [81,82]. As such, exploring the mechanism(s) underpinning the acclimation of photosynthetic apparatus to UHI, particularly the intra-specific variation, which rarely explored [82,83], is critical for urban forestry practices for a more sustainable urban ecosystem.

In this study, we selected a UHI temperature gradient to assess the PSII acclimation of *F. chinensis* (Oleaceae, Fraxinus) to a high temperature in the urban environment by our OJ fitting method. Our study showed that *F*_o_ tended to increase with the increase in land surface temperature (Figure 7d). An increase in *F*_o_ may be due to (1) a shift in Q_A_/Q_B_ equilibrium favoring Q_A_, suggesting a retarding of electron transfer from Q_A_ to Q_B_ [84,85]; (2) disconnection of LHCII from PSII RCs [86]; (3) heat-induced monomerization of LHCII trimer [87]; (4) an increase in chlorophyll content [88]; and (5) complex loss of functional and structural integrity of PSII [89]. Since *F*_o_ did not rise abruptly above a temperature threshold [90], as well as *F*_v_/*F*_m_ among sites were all greater than 0.81, both suggest no heat-induced inactivation of PSII. As such, the tendency of *F*_o_ to rise with the increase in surface temperature may be more likely related to the retarding of electron transfer from Q_A_ to Q_B_ (and beyond) and/or the increased PSII antenna as indicated by an increase in chlorophyll content, which is consistent with the significant *k*_ox_ (fitted before 0.30 ms) decrease and the increased *σ*_PSII_ (fitted before 0.30 ms). *k*_ox_ decrease is in accordance with the report by [91], who found that high temperature (35 °C) would increase the half time of flash-induced fluorescence decay and post-illumination P700^+^ re-reduction, compared to low temperatures (15–20 °C), suggesting a more reduced state of the photosynthetic electron transport chain. In fact, in contrast to heat responses [92,93,94,95], several studies found heat-acclimated leaves/waterweed showed shade-type characteristics, such as a higher chlorophyll content per unit leaf area [83,96], a lower Chl *a*:*b* ratio [97], higher SLA [97], and a lower connectivity by traditional JIP test [98,99], consistent with our results of *F*_o_, *σ*_PSII_, and *p* (fitted before 0.3 ms), which may have been associated with photo-protection of PSII. Zivcak et al. [100] hypothesized that low connectivity of shade leaves indicates less efficient transfer of excitation energy from the antenna to RCs and hence fewer electrons injected into the intersystem chain, leading to less excitation pressure and a higher photo-protection of PSII. These findings are consistent with the observation that changes in plant morphology initiated by high ambient temperature and by vegetation shade are very similar [31,32]. Although more work on the temperature responses of the diffusive and biochemical limitations [101] along the UHI gradient is warranted, while other environmental stresses combined with the temperature effect [102,103] need to be considered, this case study demonstrates that the application of fast Chl fluorescence kinetics with analytic solutions can assist us to promptly and initially identify the mechanism(s) underlying the heat stability of photosynthetic apparatus via non-intrusive methods and therefore the sustainability of urban ecosystem, in the context of global climate change and accelerated urbanization. Certainly, this method can also be useful for chlorophyll fluorescence analysis in plants subjected to different environmental stresses [84,104,105].

## 4. Materials and Methods

### 4.1. Materials

#### 4.1.1. Spinach

Measurements were carried out on mature leaves of 6-week-old spinach plants (*Spinacea oleracea* L.). Plants were grown in a greenhouse where the temperature was 18–25 °C during the day and about 14 °C at night. In the experiment, a 10-mm diameter leaf disc was measured, which was cut from the leaf sample by a piece of sharpened stainless-steel tube of appropriate diameter. A soft rubber pad was used to support the leaf during cutting.

#### 4.1.2. *Fraxinus chinensis*

Beijing is a famous megacity worldwide, centered at 116°20′ E and 39°56′ N, with a warm temperate semi-humid semi-arid monsoon climate. It has a warm temperate continental monsoon climate with an average annual temperature of 12.8 °C and an average annual precipitation was 606 mm (data from China Meteorological Administration). A typical street tree species in Beijing, *F. chinensis* (Oleaceae, Fraxinus), was used as the study object. Trees aged around 20 years (±2) were selected according to the local landscape planting data. At the same time, in order to circumvent the influence of the species source, ash trees from the same source (Baoding, China) were selected based on visits and inquiries to local landscape planning information during the preliminary sampling research.

### 4.2. Methods

#### 4.2.1. O-J Phase Fitting Model

The fluorescence yield before 2 ms (O-J, or O-I1) could be fitted by an equation based on an exciton/radical-pair model with energy transfer between photosynthetic units [1,13] as follows:

(1) $$f\left(t\right)=F_{o}+\left(F_{mj}-F_{o}\right)\times C\left(t\right)\times \frac{1-p}{1-C\left(t\right)\times p}$$

where *f*(*t*) is the fluorescence yield at time *t*, *F*_o_ is the minimal fluorescence yield when Q_A_ is fully oxidized, *F*_mj_ is the maximal fluorescence yield when Q_A_ is fully reduced at the time point J, *C*(*t*) is the fraction of closed PSII reaction centers, and *p* is Joliot’s connectivity parameter. This equation is the same as the following equation derived by Lavergne and Trissl [20]:

(2) $$f\left(t\right)=\frac{F_{mj}-O\left(t\right)\times \left[F_{mj}-F_{o}\times (1+J_{2})\right]}{1+J_{2}\times O(t)}$$

where *O*(*t*) is the fraction of open PSII reaction centers [=1 − *C*(*t*)] and *J*_2_ is a connectivity parameter related to *p* as *J*_2_ = *p*/(1 − *p*).

In Equation (1), the rate of closure of PSII reaction centers can be fitted by a differential equation (when Q_A_^−^ reoxidation is not considered, modified from Kolber et al. [1]):

(3) $$\frac{dC(t)}{dt}=\sigma_{PSII}\times I\times f_{2}\times \frac{1-C\left(t\right)}{1-C\left(t\right)\times p}$$

where *σ*_PSII_ is the PSII functional absorption cross-section, *f*_2_ is the fraction of the absorbed light partitioned to PSII, and *I* is the excitation intensity (irradiance). *I* has units of photons, m^−2^ s^−1^. Kolber et al. [1] only offered a numerical solution to the differential Equation (3); here, we provide an analytical solution by a home-made Matlab program code (Matlab, R2010b, the MathWorks, Natick, Massachusetts, USA), to yield the following function:

(4) $$C\left(t\right)=\frac{W\left(t\right)\times (1-p)}{p}+1$$

where *W*(*t*) is a Lambert W-function as follows:

(5) $$W\left(t\right)=lambertw(\frac{p\times e^{\left[\frac{\sigma_{PSII}\times I\times f_{2}\times t-p}{1-p}+\left(1-p\right)\times ln(-e^{\left(-\frac{p}{1-p}\right)})+p\right]}}{1-p})$$

where *lambertw* is the expression symbol of the Lambert W-Function; it is the converse relation of the function *f*(*W*) = *W* × e*^W^*, where *W* is any complex number and e*^W^* is the exponential function. When dealing with real numbers in the present study, the *W*_0_ branch was applied because *t* ≥ 0.

Equation (4) does not consider the Q_A_^−^ reoxidation process. At time *t*, Q_A_^−^ reoxidation occurs and the magnitude of *C*(*t*) is, therefore, a function of (1) *C*(*t*) without Q_A_^−^ reoxidation (Equation (4)), (2) the oxidation rate coefficient (*k*_ox_), and (3) the time *t*:

(6) $$C\left(t\right)=C(t)\times e^{-k_{ox}\times t}$$

where *k*_ox_ may contain several components [1], and *C*(*t*) is the Equation (4). Here, we only consider the overall oxidation rate coefficient for simplicity of fitting. Actually, we found that for nonlinear curve-fitting in the least-squares manner, multiple components cannot be distinguished.

For fitting purposes, the *F*_o_ value was the minimal reliable recorded fluorescence yield at the 50 μs time point obtained with the Handy PEA fluorimeter [12]. The value of *f*_2_ is the fraction of excitation energy partitioned to PSII. Although *f*_2_ is between 0.47 and 0.50 in spinach, poplar, rice, and cotton leaves in steady-state photosynthesis [106], its value in a dark-adapted leaf is yet to be determined. Here, *f*_2_ is assumed to be 0.5. Thus, once *F*_mj_ is known, there are three unknown variables in Equation (6), namely, *σ*_PSII_, *p*, and *k*_ox_. To derive these three parameters, we wrote a Matlab program code using the *lsqcurvefit* (), which is a nonlinear curve-fitting function based on a modified Gauss–Newton algorithm with a trust region method. It aims to guarantee the least root mean square error of prediction (RMSE) among all potential fits. The equation for RMSE is as follows:

(7) $$RMSE=\sqrt{\sum_{i=1}^{n}{\left(Y_{i}-{Y_{i}}^{\prime }\right)}^{2}\times \frac{1}{n}}$$

where *Y*_i_ is the measured values of fluorescence, *Y*_i_′ is the predicted values, and *n* is the number of data points.

#### 4.2.2. Determination of *F*_mj_ Measured by a Handy PEA Type Fluorimeter

The fitting of data obtained by a LIFT/FRR fluorimeter [1] or a PAM type fluorimeter [13,56], both of which are basically based on the exciton-radical-pair model developed by Lavergne and Trissl [20], applied a train of single-turnover flashlets or a single-turnover flash to obtain *F*_mj_ during the FRR protocols or at the J point, respectively. However, for a typical Handy PEA fluorimeter, we cannot measure *F*_mj_ at the J point. Determination of *F*_mj_ is critical not only for the requirement of model fitting but also because there is no consensus on whether Q_A_ is completely reduced at the end of the photochemical phase O-J [25]. In fact, variation in *F*_mj_ would lead to a considerable difference in the fitted *σ*_PSII_ as revealed by Figure 8b in Kolber et al. [1] (also Figure 4a in the present study). It might also be inappropriate to use the fluorescence yield at the P point to represent *F*_mj_ at the J point because of heterogenous non-photochemical quenching [26] and/or because photochemical reactions beyond Q_B_ occur after the J point [25].

**Figure A1:**
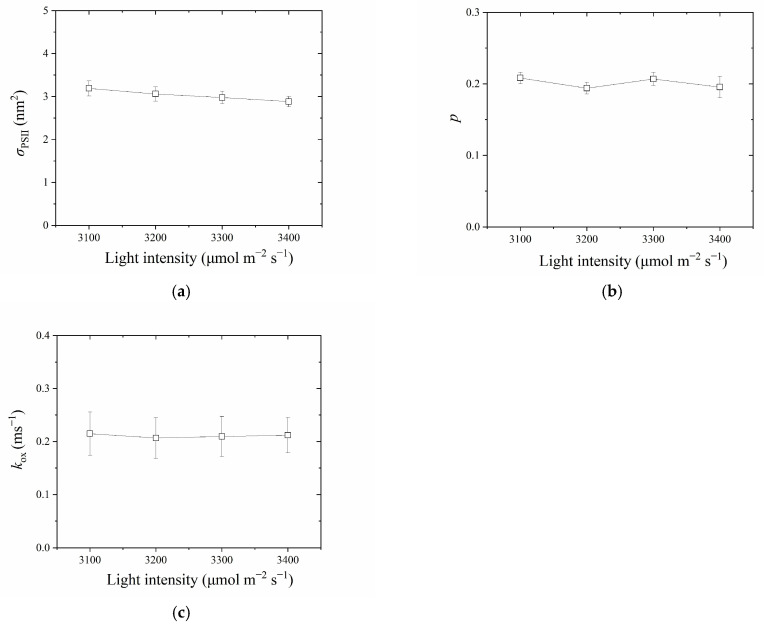
The relationship between light intensity and fitted *σ*_PSII_ (**a**), fitted *p* (**b**), and fitted *k*_ox_ (**c**). The above parameters were calculated with 1 ms data. The light intensity was 3100–3400 μmol m^−2^ s^−1^.

To determine *F*_mj_ during the O-J phase in the absence of a saturating pulse for a Handy PEA type fluorimeter, we first treated *F*_mj_ as an unknown parameter during the fitting process. However, the fitting output showed that *k*_ox_ was negative, indicating such a method is un-reliable, probably due to non-independence of *F*_mj_ to three other unknown parameters. For this reason, we sought a statistical solution to obtain *F*_mj_. It has been reported that *σ*_PSII_ is independent of the excitation light intensity during the LIFT/FRR protocols [1]. Thus, it is safe to determine *F*_mj_ by analyzing the *σ*_PSII_ variation (Figure A1). If a gradient of excitation light intensity is set up, there is a relative root mean square error of prediction (RRMSE) of *σ*_PSII_ among the OJ measurements under the corresponding excitation light intensity, given that *F*_mj_ is a priori assigned. Then, if a gradient of *F*_mj_ is a priori assigned (from the fluorescence yield at the J point to a higher unspecified value), we can expect that there exists a lowest RRMSE of *σ*_PSII_ along the gradient of excitation light intensity, at which the specified *F*_mj_ should be the best estimation of *F*_mj_ at the J point. Lower values of RRMSE indicate higher accuracy of the fitting. The RRMSE equation is

(8) $$RRMSE=\frac{\sqrt{\sum_{i=1}^{n}{\left(Y_{i}-{Y_{i}}^{\prime }\right)}^{2}\times \frac{1}{n}}}{\bar{Y_{i}}}$$

where *Ȳ*_i_ is the average of *Y*_i_.

#### 4.2.3. Measurement of *k*_ox_′ by Decay of the Chl *a* Fluorescence Yield after a Single-Turnover Flash Measured by a PAM 101-103 Fluorimeter

For a comparison with *k*_ox_ derived by the analytical solution, the parameter *k*_ox_′ can be measured by the Chl *a* fluorescence yield after a flash using a PAM 101–102–103 fluorimeter [24,107]. The decay of the flash-induced increase in Chl *a* fluorescence yield in a leaf disc was measured at room temperature using a pulse-modulated fluorimeter (PAM 101 and 103, Walz, Effeltrich, Germany). A single-turnover flash (full width at half height 6 μs) was given by an XE-ST xenon flash lamp unit (model XF-103, Walz). Weak modulated light (450 nm) was automatically switched to 100 kHz when a single-turnover flash was given. Data acquisition (time constant 15 μs) was achieved by home-built equipment and a computer program [108]. Twenty successive flashes were given every 5 s and the signals were averaged. The flash overload artifacts (the initial 3–4 points) after a flash were discarded before kinetic fitting. The fitting follows a first-order reaction, with *k*_ox_′ (the measured Q_A_ oxidation rate coefficient, ms^−1^) and the amplitude (*A*) as the unknowns being sought. The equation to fit the decay of the flash-induced increase in Chl *a* fluorescence yield is

(9) $$y=A*e^{-{k_{ox}}^{\prime }*t}$$

where *y* is the fluorescence yield and *t* is the time (ms). *k*_ox_′ and *A* were obtained by the software Origin (Version 7.0, Microcal Software Inc., Northhampton, MA, USA) based on the Levenberg–Marquardt method. *k*_ox_′ and *A* were a priori assigned initial values; after a number of iterations both would tend towards stable values. The value of *k*_ox_′ should be close to that of *k*_ox_ obtained by fitting with Equation (6) if the *F*_mj_ estimated by the statistical method is reliable under single-turnover situations.

#### 4.2.4. DCMU Treatment

Spinach plants were placed in darkness for about 1 h before treatment of leaf discs with 3-(3,4-dichlorophenyl)-1,1-dimethylurea (DCMU). Leaf discs of 10-mm diameter were prepared and floated on a 10-mL DCMU solution (the DCMU concentration was 200 μM and the solution contained 1% ethanol, which was used to dissolve the DCMU). For the controls, leaves were left untreated on distilled water. The treatment with DCMU was carried out for about 14 h in complete darkness [38]. Following the treatment, leaves were removed from the DCMU solution (still in darkness) and gently blotted before measurements.

**Figure A2:**
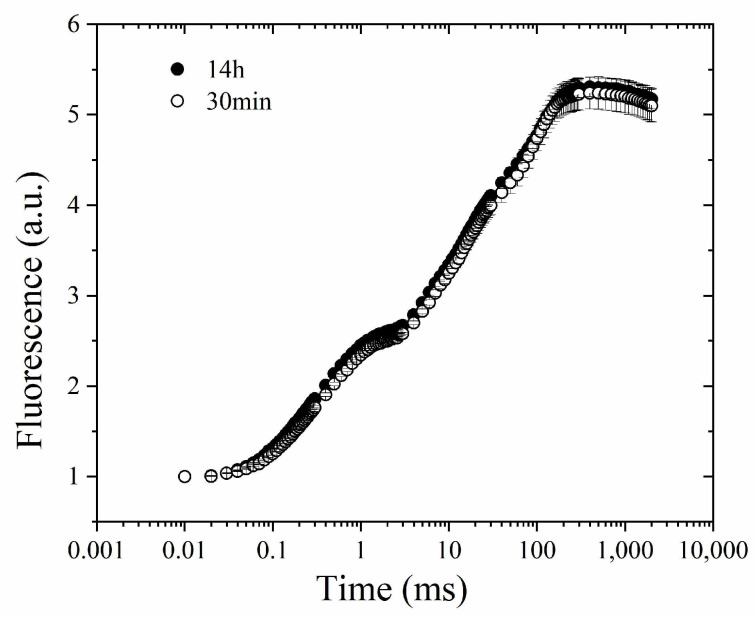
The OJIP curves obtained after 30 min and 14 h dark adaptation.

Chl *a* fluorescence transients were measured using a Handy plant efficiency analyzer (Hansatech Instruments Ltd., King’s Lynn, Norfolk, UK) with an actinic light of 3100, 3200, 3300, or 3400 μmol m^−2^ s^−1^ by the method described by Strasser et al. [12], with a 30-min darkness interval between each light for the same leaf disc for control. Actually, we found that the OJIP curves obtained by 30 min and 14 h dark adaptation were very similar, as shown in Figure A2. For DCMU treatment, different leaf discs were used. All the fluorescence transients were recorded within a time scan from 10 μs to 1 s with a data acquisition rate of 100,000 readings per second for the first 2 ms and 1000 per second after 2 ms, after the leaf had been pre-darkened for 30 min. *F*_J_ is the fluorescence yield at 2 ms.

#### 4.2.5. UHI Sampling and Measurement

Land surface temperature is an index to characterize the degree of urban heat island in a region [109]. In this study, we downloaded Landsat 8 data from China Geospatial Data Cloud open-source data and used the land surface temperature inversion system made by Ren et al. [110,111] to obtain the annual average land surface temperature (LST) in Beijing.

**Figure A3:**
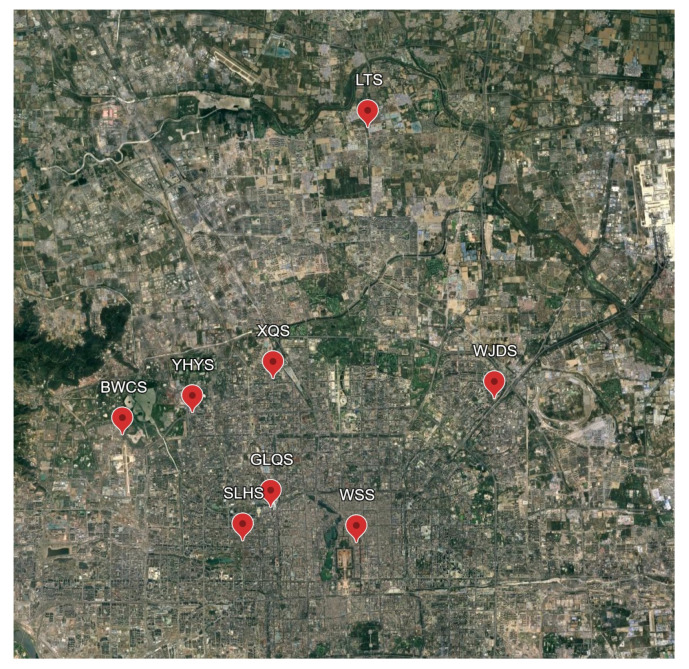
Geographical distribution map of the eight experimental sites in Beijing (source: Google Earth). LTS, Litang Street; XQS, Xueqing Street; WJDS, Wangjingdong Street; YHYS, Yiheyuan Street; BWCS, Beiwucun Street; GLQS, Gaoliangqiao Street; SLHS, Sanlihe Street; WSS, Wusi Street. In this study, we used a land surface temperature inversion algorithm to obtain the annual average land surface temperature (LST).

Eight sampling sites with different urban heat island intensities (represented by LST) were selected. The sampling environment avoided street valleys and the shading of tall buildings, with no large lake or rivers within 10 km from the center of the sampling area. The hardened surface type of the streets was asphalt and the samples were away from street lighting. This would basically ensure the relative consistency of the sampling environmental conditions other than LST. The positions of the eight sampling sites are shown in Figure A3.

On sunny days in July 2023, three trees at each sampling site were selected. A total of twelve mature and healthy leaf samples were randomly collected from sun-exposed branches (mean height: 4.5 m) from three trees at each site at predawn. Sampled leaves were immediately wrapped with wet gauze, put in a dark box, and transferred to the laboratory. Chlorophyll fluorescence was recorded on these >30 min dark-adapted leaves by a chlorophyll fluorimeter Handy PEA (Hansatech Instruments Ltd., King’s Lynn, UK) by the method aforementioned.

## 5. Conclusions

In the present study, an analytical solution to the differential equation of Q_A_ reduction kinetics has been presented in order to minimize uncertainties of parameter estimation, if any. We also sought a statistical solution to find *F*_mj_ for a Handy PEA-type fluorescence meter based on the prediction by the “exciton/radical-pair equilibrium” model. We found that the PSII functional absorption cross-section, connectivity, and Q_A_ oxidation rate coefficient showed dynamic changes during the dark–light transition period. Such dynamic changes can potentially reconcile the dispute on whether there exists energy connectivity between PSIIs. We hence suggested that the estimated PSII excitonic connectivity and functional absorption cross-section can only be compared across species or treatments when other pathways of energy dissipation are not too dominant/variable. For a Handy PEA-type fluorimeter, we recommend that the fitting of data points up to 0.3 ms of illumination is a good option. We also conducted a case study on the UHI effect on the heat stability of PSII by our method and showed that high temperature-acclimated leaves had a higher *σ*_PSII_, lower *k*_ox_, and a tendency of lower *p* towards more shade-type characteristics. This demonstrates that the application of our method can assist us in promptly identifying the mechanism(s) underlying the heat stability of photosynthetic apparatus via a non-intrusive method and, therefore, the sustainability of the urban ecosystem, in the context of global climate change and accelerated urbanization.

## Data Availability

The raw data supporting the conclusions of this article will be made available by the authors, without undue reservation.

## Abbreviations

Chl	Chlorophyll
C(*t*)	The fraction of closed PSII reaction centers
DCMU	3-(3,4-Dichlorophenyl)-1,1-dimethylurea
FI	Fluorescence induction
FRR	Fast repetition rate
*F*_m_	Dark-acclimated maximal fluorescence yield
*F*_o_	Dark-acclimated minimal fluorescence yield
*F*_mj_	The maximum yields of chlorophyll fluorescence as induced by a single-turnover saturating flash at/before the J point
F_v_/F_m_	Maximum quantum yield of primary photosystem II photochemistry
f(t)	Fluorescence yield at time *t*
*f*_2_	The fraction of the absorbed light partitioned to PSII
J	Sigmoidicity/connectivity parameter
J_2_	Connectivity parameter related to *p* as *J*_2_ = *p*/(1 − *p*).
*k*_ox_	The rate coefficient for Q_A_ oxidation output by the OJ fitting (ms^−1^)
*k*_ox_′	The measured rate coefficient for Q_A_ oxidation (ms^−1^)
LHCII	Light-harvesting complex associated with PSII
LIFT	Light-induced fluorescence transients
LST	Land surface temperature
I	Excitation intensity (irradiance) (photons m^−2^ s^−1^)
NPQ	Non-photochemical quenching
O-J-I-P	Fluorescence rise on a dark-to-light transition from a minimum value O via the intermediate steps J and I to the maximum value P, which is *F*_m_ if the light is saturating
O(*t*)	The fraction of open PSII reaction centers [= 1 − C(*t*)]
p	The connectivity among PSII complexes
PEA	Plant efficiency analyzer
PS II, PS I	Photosystem II, photosystem I, respectively
P680, P700	PSII and PSI reaction center chlorophyll dimer, respectively
Q_A_, Q_B_, PQ	Primary and secondary quinone electron acceptors of PSII and free plastoquinone, respectively
RC	Reaction center
RMSE	Root mean square error
RRMSE	Relative root mean square error
STF	Single turn-over flash
UHI	Urban heat island
*W*(*t*)	Lambert W-Function
W	Any complex number
*σ*_PSII_	The functional absorption cross-section of PSII (nm^2^)

## Appendix A

(1) Two-channel nonlinear split-window algorithm

(A1) $$T_{s}=b_{0}+(b_{1}+b_{2}*\frac{1-\varepsilon }{\varepsilon }+b_{3}*\frac{∆\varepsilon }{\varepsilon^{2}})*\frac{T_{i}+T_{j}}{2}+(b_{4}+b_{5}*\frac{1-\varepsilon }{\varepsilon }+b_{6}*\frac{∆\varepsilon }{\varepsilon^{2}}\frac{T_{i}+T_{j}}{2}+b_{7}*{{(T}_{i}+T_{j})}^{2}$$

where T_i_ and T_j_ are the TOA brightness temperatures measured in channels i (~11.0 μm) and j (~12.0 µm), respectively; ε is the average emissivity of the two channels (i.e., ε = 0.5 [ε_i_ + ε_j_]), whilst Δε is the channel emissivity difference (i.e., Δε = ε_i_ − ε_j_); b_i_ (i = 0, 1 … 7) and can be obtained from laboratory data, atmospheric parameter data, and simulated datasets of atmospheric radiation transfer equation. In order to improve the inversion accuracy, b_i_ will depend on atmospheric water vapor (wv) [108].

(2) Water Vapor Retrieval Technique

The split-window covariance–variance ratio (SWCVR) method is used to retrieve atmospheric water vapor. Assuming that the atmosphere is unchanged over the neighboring pixels where the land surface temperature and emissivities change, the SWCVR method relates wv to the ratio of the upward transmittances of two thermal infrared bands (approximately 11 μm and 12 μm), while the transmittance ratio can be calculated from the brightness temperatures of the two bands at the top of the atmosphere (TOA). Considering N adjacent pixels, wv in the SWCVR method is estimated as

(A2) $$wv=a+b*(\tau_{j}/\tau_{i})+c*{\left(\tau_{j}/\tau_{i}\right)}^{2}$$

(A3) $$\tau_{j}/\tau_{i}\approx R_{ji}=\sum_{k=1}^{N}\left(T_{i,k}-{\bar{T}}_{i})(T_{j,k}-{\bar{T}}_{j}\right)/\sum_{k=1}^{N}{\left(T_{i,k}-{\bar{T}}_{i}\right)}^{2}$$
